# Effectiveness and cost-effectiveness of daily all-over-body application of emollient during the first year of life for preventing atopic eczema in high-risk children (The BEEP trial): protocol for a randomised controlled trial

**DOI:** 10.1186/s13063-017-2031-3

**Published:** 2017-07-21

**Authors:** Joanne R. Chalmers, Rachel H. Haines, Eleanor J. Mitchell, Kim S. Thomas, Sara J. Brown, Matthew Ridd, Sandra Lawton, Eric L. Simpson, Michael J. Cork, Tracey H. Sach, Lucy E. Bradshaw, Alan A. Montgomery, Robert J. Boyle, Hywel C. Williams

**Affiliations:** 1 Centre of Evidence Based Dermatology, University of Nottingham, Nottingham, UK; 2 Nottingham Clinical Trials Unit, University of Nottingham, Nottingham, UK; 3 Skin Research Group, School of Medicine, University of Dundee, Dundee, UK; 4 Department of Dermatology, Ninewells Hospital and Medical School, Dundee, UK; 5 School of Social and Community Medicine, University of Bristol, Bristol, UK; 6 The Rotherham NHS Foundation Trust, Moorgate Road, Rotherham, UK; 7 Department of Dermatology, Oregon Health and Science University, Portland, OR USA; 8 Department of Infection and Immunity, Sheffield, UK; 9 Health Economics Group, Norwich Medical School, University of East Anglia, Norwich Research Park, Norwich, UK; 10 Section of Paediatrics, Imperial College London, Wright Fleming Building, London, UK

**Keywords:** Protocol, Randomised controlled trial, Eczema, Atopic dermatitis, Prevention, Emollient, Barrier enhancement, Filaggrin, Core outcomes

## Abstract

**Background:**

Atopic eczema (AE) is a common skin problem that impairs quality of life and is associated with the development of other atopic diseases including asthma, food allergy and allergic rhinitis. AE treatment is a significant cost burden for health care providers. The purpose of the trial is to investigate whether daily application of emollients for the first year of life can prevent AE developing in high-risk infants (first-degree relative with asthma, AE or allergic rhinitis).

**Methods:**

This is a protocol for a pragmatic, two-arm, randomised controlled, multicentre trial. Up to 1400 term infants at high risk of developing AE will be recruited through the community, primary and secondary care in England. Participating families will be randomised in a 1:1 ratio to receive general infant skin-care advice, or general skin-care advice plus emollients with advice to apply daily to the infant for the first year of life. Families will not be blinded to treatment allocation. The primary outcome will be a blinded assessment of AE at 24 months of age using the UK Working Party Diagnostic Criteria for Atopic Eczema. Secondary outcomes are other definitions of AE, time to AE onset, severity of AE (EASI and POEM), presence of other allergic diseases including food allergy, asthma and hay fever, allergic sensitisation, quality of life, cost-effectiveness and safety of the emollients. Subgroup analyses are planned for the primary outcome according to filaggrin genotype and the number of first-degree relatives with AE and other atopic diseases. Families will be followed up by online and postal questionnaire at 3, 6, 12 and 18 months with a face-to-face visit at 24 months. Long-term follow-up until 60 months will be via annual questionnaires.

**Discussion:**

This trial will demonstrate whether skin-barrier enhancement through daily emollient for the first year of life can prevent AE from developing in high-risk infants. If effective, this simple and cheap intervention has the potential to result in significant cost savings for health care providers throughout the world by preventing AE and possibly other associated allergic diseases.

**Trial registration:**

ISRCTN registry; ID: ISRCTN21528841. Registered on 25 July 2014.

**Electronic supplementary material:**

The online version of this article (doi:10.1186/s13063-017-2031-3) contains supplementary material, which is available to authorized users.

## Background

Atopic eczema (syn. atopic dermatitis or eczema) [1] is a very common chronic skin problem affecting 16 to 30% of UK children and around 20% worldwide [2, 3]. Atopic eczema usually starts in infancy and around 40% of cases persist into adulthood, especially those with early onset and widespread disease [4]. The family impact of caring for a child with moderate or severe atopic eczema is greater than that in caring for children with type 1 diabetes mellitus, mainly due to sleep deprivation, employment loss, time to care for atopic eczema and financial costs [5].

Children with atopic eczema, especially those with severe disease, are at increased risk of also developing other allergic (immunoglobulin E (IgE)-mediated) diseases including food allergy, allergic asthma and allergic rhinitis (hay fever) [6–8]. Eczema is often the first manifestation of the so-called ‘atopic march’, in which a child progresses from atopic eczema to food allergy, asthma and allergic rhinitis later in life [9, 10]. Together, these atopic diseases are the most common chronic diseases of childhood and represent a major financial burden to the UK National Health Service (NHS), with direct costs estimated at over £1 billion per annum in 2004 [11]. There is a strong association between atopic eczema during infancy and the risk of food allergy, with the highest prevalence of food allergy reported in early onset and severe atopic eczema. A causal link has been proposed [12], and this is supported by evidence from mouse and human studies showing that sensitisation to some foods can occur across a defective skin barrier [13, 14].

Atopic eczema is highly heritable and shows strong familial clustering. Prevalent mutations in the gene encoding filaggrin (*FLG*), a key skin-barrier protein, represent the strongest and most consistent known genetic risk factor for atopic eczema [15, 16].

Emollient (moisturiser) therapy is intended to improve the barrier function of the skin. An emollient provides lipids to the stratum corneum, which in turn, improves skin hydration by trapping water. Emollients also help to prevent inflammation caused by external irritants as evidenced by their benefit in preventing irritant occupational hand eczema [17]. In premature babies, emollients have been shown to reduce the incidence of skin inflammation [18], and in people with atopic eczema, to reduce flares of atopic eczema (secondary prevention) [19].

Primary prevention is a highly desirable goal in a complex chronic disease like atopic eczema with no cure. If primary prevention of atopic eczema using a strategy of early skin-barrier enhancement with simple low-cost emollients is effective, it would represent a significant cost saving for health care providers through reduced treatment and appointment costs. Further cost savings would result if early skin-barrier enhancement prevents sensitisation and associated food allergy, asthma or allergic rhinitis [11]. Even if the frequency of atopic eczema cannot be significantly reduced, a shift in the severity distribution of atopic eczema towards milder cases could improve quality of life and reduce carer burden and health care provider costs.

We carried out pilot studies to inform the design of this trial. A functional mechanistic study provided evidence for the choice of emollients for this trial and showed they are not associated with any harm to the skin barrier [20]. A multicentre, randomised controlled pilot trial of 124 families showed that families found the intervention acceptable and thus it would be feasible to conduct a larger trial of emollients for the prevention of atopic eczema [21]. Clinical outcomes showed that infants in the emollient group had a significantly reduced risk of developing atopic eczema by 6 months of age compared to those in the control group (43% versus 22%, respectively, relative risk, 0.50; 95% CI, 0.28–0.90; *P* = .017). A further trial from Japan that included 118 infants showed similar results, with 32% fewer infants in the emollient group developing atopic eczema [22]. This large, pragmatic trial (the Barrier Enhancement for Eczema Prevention (BEEP) trial) described here is now required to confirm the results seen in these small, short-term trials and to establish the long-term effects of emollients for preventing atopic eczema and associated allergic diseases.

This is an abridged protocol based on protocol version 5.0 dated 26 October 2016. The full protocol is available on the trial website [23]. This protocol adheres to the Standard Protocol Items: Recommendations for Interventional Trials (SPIRIT) recommendations for interventional trials and the SPIRIT Checklist is included (see Additional file 1).

### Objectives

- The main objective is to determine whether applying emollient daily to the entire body surface area for the first year of life can prevent atopic eczema in high-risk children
- Other objectives are to investigate:
  - ○ Whether emollients can delay the onset and/or reduce the severity in those who develop atopic eczema
  - ○ Whether emollients can prevent other allergic diseases developing
  - ○ The safety and cost-effectiveness of the prevention strategy
  - ○ The role of *FLG* genotyping as possible stratifier of response to emollient intervention

## Methods/design

### Trial design

**Fig. 1:**
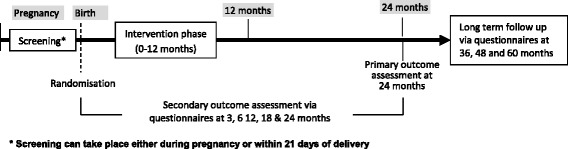
Schematic diagram showing the trial design and duration for participating families

**Fig. 2:**
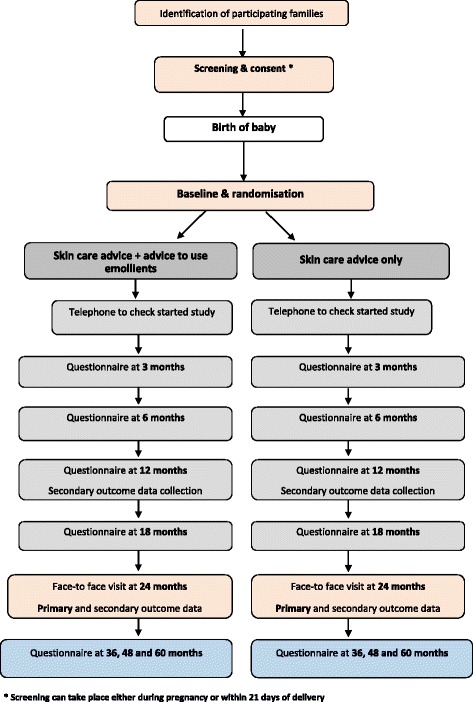
Flowchart indicating participant flow through the trial

**Fig. 3:**
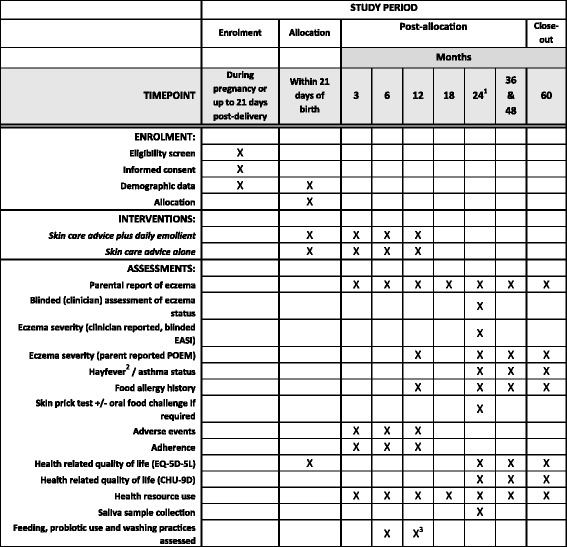
Standard Protocol Items: Recommendations for Interventional Trials (SPIRIT) figure showing important events in the trial and their respective time points. EASI - Eczema Area and Severity Index, POEM Patient Oriented Eczema Measure, EQ-5D-5 L – EuroQol Five Dimension, CHU-9D – The Child Health Utility. ^1^The 24 month is a face-to-face visit from the research nurses blinded to treatment allocation. ^2^Hayfever status assessed at 60 month only. ^3^Washing practices only

This is a randomised, controlled, two-arm (skin-care advice plus emollient versus skin-care advice alone), parallel-group, multicentre, assessor-blind trial (Fig. 1). It is a pragmatic design in which investigators have no scheduled contact with the families between randomisation and the 24-month visit in order to limit any influence on adherence, minimise the risk of un-blinding and keep the research nurse resources required to a reasonable level. All other contact is with the trial coordinating centre (Figs. 2 and 3). Screening for eligibility and the consent process is carried out either antenatally or shortly after delivery. Further eligibility checks are carried out post delivery prior to randomisation which takes place within 3 weeks of delivery.

### Setting

The trial is recruiting families across England. Identification is via primary care (mailshot invitations to participate), secondary care (through antenatal care, dermatology clinics and posters), and in the community via direct advertising. Recruiting sites are mainly secondary care centres, with a smaller number of primary care centres involved. A list of recruiting centres can be found in the acknowledgements.

### Participants

Infants at high-risk of developing atopic eczema, defined as having a first-degree relative with parent-reported physician-diagnosed atopic eczema, asthma or allergic rhinitis, are eligible for the trial. The infant must be no more than 21 days old at the point of randomisation, the mother must be aged at least 16 years and the consenting adult must be able to understand English. Infants are not eligible if they are born prior to 37 weeks’ gestation, have a severe skin condition at time of randomisation that would make it difficult to assess the skin for signs of atopic eczema or that would preclude the use of emollients (such as dystrophic epidermolysis bullosa, ichthyosiform erythroderma or lamellar ichthyosis), or if they have a serious health issue that would make participation difficult for the family (e.g. neonatal sepsis or major congenital abnormalities). Only the first born of any multiple births will be randomised.

### Intervention

Both groups receive general skin-care advice to avoid soap and bubble bath, use mild cleansers and shampoos that have been specifically designed for babies, and avoid using baby wipes based on NICE guidance on infant skin care, July 2006 [24]. The use of bath oils is also discouraged unless directed to do so by a health care professional. Furthermore, the intervention group receives additional advice to apply emollient daily for the first year of life and are supplied with emollient free of charge. Parents are able to choose between Doublebase Gel® (Dermal Laboratories Ltd.) and Diprobase Cream® (Merck Sharp & Dohme Ltd.) and can switch emollient during the trial should they wish to. These emollients were chosen because they have a similar formulation to many emollient creams used in the UK, which would mean that the results of this trial would be applicable to a range of emollients with similar formulation and not restricted to the two emollients used in this trial. Emollients containing sodium lauryl sulphate (SLS) as an emulsifier were specifically excluded because they have been shown to have negative effects on the skin barrier [25]. The skin-care advice for both groups is sent to families within 2 days of randomisation in the form of a booklet and a short online video. These materials will be available as part of the final trial report.

The intervention group advice includes how to apply the emollient; at least once daily all over the infant (the scalp can be avoided) and the emollient should always be applied bathing, even if it has previously been applied that day. Parents are advised to start the emollient as soon as possible after birth and definitely within 3 weeks and the start date of emollient usage is recorded by the trial coordinating centre during a routine 2-week post-randomisation phone call. Emollient is continued until the child is 1 year old and with parental reported usage collected at 3, 6 and 12 months. The trial coordinating centre keeps a record of the quantity of emollient provided to each participating family.

If the infant develops skin problems during the trial, parents are advised to seek medical help in the usual way. Any eczema that develops will be treated as per normal practice regardless of treatment group allocation.

### Primary outcome

The primary outcome is a diagnosis of atopic eczema at 24 months, defined as meeting the UK Working Party Diagnostic Criteria for Atopic Eczema [26] which assesses signs and symptoms present over the past year. The criteria will be applied by a trained research nurse blinded to treatment allocation. Applying these criteria when the infants are 24 months old will detect atopic eczema that has developed between the ages of 12 and 24 months and will, therefore, exclude transient eczematous rashes common in the first year of life that are often not true atopic eczema. Measuring the outcome at 24 months also ensures that any observed effect on reducing atopic eczema prevalence is a true preventative effect rather than a treatment effect of the emollient caused by shifting those with mild atopic eczema into the subclinical range.

### Secondary outcomes

The secondary outcomes relate to outcomes up to and including the 24-month time point:

1. Presence of atopic eczema between birth and 24 months defined as:
  - Parental report of a clinical diagnosis of atopic eczema
  - Parental completion of UK Working Party Diagnostic Criteria for Atopic Eczema at 12 and 24 months (Additiona﻿l file 2)
2. Presence of visible atopic eczema at 24 months (assessed by a trained research nurse blinded to treatment allocation)
3. Time to onset of atopic eczema:
  - First parental report of a clinical diagnosis of atopic eczema
  - First topical corticosteroid and/or immunosuppressant prescription for atopic eczema
4. Severity of atopic eczema:
  - Eczema Area and Severity Index (EASI) (Additional f﻿ile 3) at 24 months and
  - Patient-oriented eczema measure (POEM) (Additional file 4) at 12 and 24 months. EASI and POEM are the core outcome instruments recommended by the Harmonising Outcome Measures for Eczema (HOME) for measuring clinician-reported signs and patient-reported symptoms respectively [27, 28] and are both well validated [29, 30]. The EASI will be completed by a trained research nurse who is blinded to treatment allocation
5. Presence of other allergic diseases:
  - Parental-reported wheezing, allergic rhinitis and food allergy symptoms, and parental report of a clinical diagnosis of food allergy between 12 and 24 months
  - Allergic sensitisation at 24 months to any of the following common allergens: milk, egg, peanut, cat, grass pollen, house dust mite
  - Confirmed diagnosis of food allergy at 24 months to milk, egg, and/or peanut derived from a combination of parental report, allergic sensitisation and (if required) food challenge
6. Health-related quality of life:
  - Child quality of life using the Child Health Utility 9D (CHU-9D) at 24 months
  - Parental quality of life measured using the EuroQol-5D-5 L (EQ-5D-5 L) at baseline and 24 months
7. Health economic outcomes:
  - Disease-related health care resource use
  - Cost-effectiveness and cost-utility at 24 months (combining health resource use and health-related quality of life outcomes). If significantly more effective than usual care at 24 months, a longer-term economic model from birth to 16 years we be developed

### Safety outcomes

Safety of the emollient determined from the number of skin infections and infant slippage incidents related to emollient use during the intervention period.

### Tertiary outcomes

1. Parental opinion that their child has atopic eczema at any point during the trial.
  Data on tertiary outcomes 2–5 are collected at 36, 48 and 60 months:
2. Parental report of a clinical diagnosis of atopic eczema
3. Severity of atopic eczema (POEM)
4. Presence of other atopic diseases:
  - Parental-reported wheezing, allergic rhinitis and food allergy symptoms
  - Parental report of a clinical diagnosis of asthma, allergic rhinitis or food allergy
  - Health-related quality of life (child; CHU-9D and parent; EQ-5D-5 L)
5. Health economic outcomes:
  - Disease-related health care resource use
  - Cost-effectiveness and cost-utility (combining health resource use and health-related quality of life outcomes)

### Randomisation, allocation concealment and blinding

The randomisation schedule (1:1 ratio) is based on a computer-generated pseudo-random code using random permuted blocks of randomly varying size. It was created by the Nottingham Clinical Trials Unit (NCTU) and is held on a secure University of Nottingham server. During the trial, access to the sequence is confined to the IT programmer at NCTU. Randomisation is stratified by recruiting centre and number of immediate family members (parents or siblings) with atopic disease (1, 2, or more than 2). Recruiting centre staff randomise participants via a web-based randomisation system developed and maintained by NCTU. Recruiting centre staff are not sent the results of the randomisation. It is not possible to blind parents as to which group they are in, but the trial nurses conducting the skin examination at the 2-year visit are blinded to treatment allocation.

### Study procedures and data collection

The screening and consent visit take place either in the family home or at the recruiting site, depending on parent preference, and consent obtained by the trial nurse (Figs. 2 and 3). Separate optional consent is obtained for the *FLG* genotyping, skin-prick testing and food challenges. Randomisation takes place after the infant is born and post-birth eligibility checks are completed. Parents are sent questionnaires online (or paper if requested) at 3, 6, 12, 18, 36, 48 and 60 months to collect outcome data. There is a second face-to-face visit with the trial nurse at 24 months, again in the family home or at the recruiting centre depending on parent preference. At this 24-month visit the blinded trial nurse conducts the skin examination for the diagnostic criteria and the EASI, completes the questionnaires, and (where consent is given) takes the saliva sample and carries out the skin-prick test. A summary of the data collection can be found in Table 1: The schedule of trial assessments. The trial nurses are fully trained in the diagnosis of atopic eczema and carrying out the EASI.

Saliva samples are collected via the child spitting into a pot or swabs taken from the inside of the child’s cheek and sent to the Centre for Dermatology and Genetic Medicine, University of Dundee for deoxyribonucleic acid (DNA) extraction by standard techniques and *FLG* genotyping for the most prevalent null mutations in the white European population (2282del4, R501X, S3247X and R2447X) according to published protocols [31]. The skin-prick testing is carried out in line with the British Society for Allergy and Clinical Immunology procedures [32] and the following allergens are tested; grass pollen mix, dust mite and cat (Allergopharma, Germany), peanut (Inmunotek, Spain), fresh skimmed cow’s milk and fresh chicken egg. Positive (1% histamine) and negative (0.9% saline) controls will also be used (Allergopharma, Germany). The trial nurses are fully trained in conducting skin-prick tests and emergency procedures in the highly unlikely event of any serious allergic reactions. Participants with a positive skin-prick test or history suggestive for food allergy in whom further investigation is required for a diagnosis of food allergy to be made are invited for a supervised oral food challenge conducted by experienced allergy nurses following standard procedures who are blinded to treatment allocation. The presence of a clinical reaction is determined using modified PRACTALL and iFAAM criteria [33, 34].

A methodological two-by-two factorial substudy is nested within this trial to investigate the effectiveness of interventions designed to improve rates of follow-up data collection. The interventions are SMS text message notifications that the questionnaires will be sent by email or post the following day versus no text message and the £10 inconvenience voucher either sent to parents either before the visit, or given at the 24-month visit. Full details can be found in the Studies Within A Trial (SWAT) registry [35].

Small tokens of appreciation and birthday cards are sent to all participating families throughout the trial to promote retention, and the trial coordinating centre will make every effort to keep the parents contact details up to date throughout the trial. When questionnaires are not completed online or by post, families are telephoned by trial coordinating centre staff. Families wishing to withdraw from the intervention are encouraged to continue to provide data on the main outcomes, with particular importance placed on the 24-month face-to-face visit where the primary outcome data is collected. Where a face-to-face visit is not possible (e.g. the family have moved abroad) key outcomes are collected via remote means (e.g. telephone, text, email or post).

Data will be entered directly onto the database by the trial nurses or parents via online questionnaires. All data are treated confidentially and held on a secure University of Nottingham server with restricted and password-protected access. Questionnaires and other data collection forms will be available as part of the final trial report.

### Sample size calculation

The sample size calculation was carried out using Stata [36, 37]. A total of 1282 infants are required to detect a relative reduction of 30% in the intervention group in the number of infants who developed atopic eczema in the previous year (between 12 and 24 months of age) at the 5% significance level (two-sided) with 90% power based on an expected rate of 30% in the control group and allowing for 20% attrition at 24 months. This relative reduction is considered a conservative estimate; results of the pilot study showed a 50% reduction in atopic eczema at 6 months (43% developed atopic eczema in the control group (*n* = 55) compared to 22% in the emollient group (*n* = 53), 95% CI 0.28 to 0.9) [21]. The effect size is anticipated to be lower in this main trial than in the pilot due to the more pragmatic trial design and the longer-term outcome assessment.

Recruitment began in November 2014 and ended in November 2016. A sample size review by the TSC took place after 21 months of recruitment to check the atopic eczema rate in the control group and attrition. As recruitment had progressed better than expected, the TSC advised that no additional families should be consented to the trial, but those who had already consented to the trial should continue to be randomised. The total number randomised at that point was expected to be approximately 1400.

### Planned analysis

Analysis of the primary, secondary and safety outcomes will be performed when all the 24-month data have been collected. The longer-term tertiary outcomes will be analysed once data collection is complete for the 60-month follow-up.

The main approach to all analyses will be to analyse participants as randomised (intention-to-treat), regardless of adherence with allocation and without imputation for missing data. All analyses will be carried out using Stata/SE 13 or above [36].

The primary outcome will be analysed using a generalised linear model adjusting for stratification variables. The difference between the two groups will be summarised using a relative risk with 95% confidence intervals. Sensitivity analyses will be performed using multiple imputation for missing outcomes by including any prognostic variables showing a baseline imbalance in the model and accounting for actual emollient use. Analyses of secondary and long-term outcomes will use appropriate regression models depending on the type of outcome and differences between the two groups summarised with 95% confidence intervals. Descriptive analysis of safety endpoints will be presented both according to randomised group and according to actual emollient use in the two groups. Planned subgroup analyses will be conducted by including an interaction term in the regression analysis for the primary outcome according to: (1) whether an individual is *FLG-*wild-type genotype or whether they have one or two of the screened *FLG* null mutations, (2).the number of immediate family members with atopic disease and (3) the number of immediate family members with atopic eczema.

Full details of the analyses and potential sensitivity analyses for the food allergy/sensitisation outcomes will be documented in the Statistical Analysis Plan prior to any analysis and made publicly available on the trial website [23].

### Economic evaluation

Economic evaluations will be conducted to estimate the cost-effectiveness of the intervention from an NHS perspective in the short term (24 months within trial analysis), medium term (60 months within trial analysis) and, if appropriate, longer term (birth to 16 years using a model-based analysis). For the within trial analyses, the incremental cost per atopic eczema case prevented, incremental cost per quality-adjusted life year (QALY) based on CHU-9D (parental-proxy reported), and incremental cost per QALY based on main carer own health-related quality of life (EQ-5D-5 L) will be estimated. An incremental cost-effectiveness analysis will be performed using accepted methods with data reported in a disaggregated way [38–41]. Analysis of uncertainty will follow recommended practice with results presented as cost-effectiveness acceptability curves [42, 43]. If the intervention is significantly more effective at 24 months than normal practice, a longer-term economic model taking an NHS perspective will be developed for the economic costs and benefits of the intervention for a single birth cohort from birth to 16 years using trial data, within-trial cost-effectiveness analyses using data collected during the first 24 months and at 36, 48 and 60 months, other published data, expert opinion and population datasets (where appropriate and available).

### Trial oversight

Trial oversight is provided by the Trial Steering Committee (TSC) which comprises an independent chair and three independent members (including one patient representative). Further details can be found in the ‘Acknowledgements’ section. A separate Data Monitoring Committee is not required due to the very low risk associated with the intervention, so this function will be covered by the TSC.

## Discussion

This trial of up to 1400 infants at high-risk of developing atopic eczema will investigate whether daily, all-over application of emollients (of a defined type of formulation) from birth for the first year of life can prevent atopic eczema developing by 2 years of age, and whether any such preventive effects are maintained or are reduced up to the age of 5 years. It will also show whether the intervention can delay the onset of atopic eczema, or whether it alters the severity distribution towards milder disease. The effect on any other atopic conditions associated with atopic eczema (allergic rhinitis, asthma and food allergy) will also be assessed. By assessing the *FLG* genotype of participants, the extent to which any effect of emollients can be modified by filaggrin haploinsufficiency will also be determined. Any differential effect according to *FLG* genotype could be used for a personalised approach to the future use of emollient for the prevention of atopic eczema.

We have taken a pragmatic approach to all aspects of this trial including a limited number of exclusion criteria, interfering as little as possible in the use of the emollient, minimal follow-up and an intention-to-treat analysis. However, as with all pragmatic trials, there are some limitations. Families participating in the trial are likely to be more motivated to use the emollients than the average population. Even so, we may observe a null result due to poor adherence because of the pragmatic nature of the advice to use emollients, whereas the intervention may be effective under conditions that enhance adherence. We have included only families whose child is at higher risk of developing eczema due to family history of atopy, although other groups are planning trials in unselected-for risk populations. Also, we have only offered two emollients in this trial, but there are many others commonly prescribed or purchased by parents. If the trial demonstrates that this relatively cheap and simple intervention is effective in preventing atopic eczema, uptake of the intervention will result in reduced costs for health care providers such as the UK National Health Service. Furthermore, if any effect is extended to the prevention of other atopic conditions then the cost savings could be significantly greater.

The results of the trial will be submitted for publication in peer-reviewed journals and the National Institute for Health Research (NIHR) journal series, and disseminated to health care providers and professional groups including health visitors, midwives, GPs, dermatologists and commissioners. Health care providers and professionals will then have the evidence required to make funding and health care decisions about emollients for preventing atopic eczema. We will inform all trial participants of the results of the main 2-year analysis and 5-year analysis and we will also post our results on the trial register. A variety of media outlets will be used to disseminate the results directly to pregnant women and new parents. Parents are often anxious to know whether their children will develop atopic eczema, especially those with experience of atopic eczema, and are keen to know what they can do minimise the risk. The results of this trial will help inform families whether emollients are an effective prevention strategy.

Other trials of emollients and related interventions for the prevention of atopic eczema are being undertaken throughout the world, and we have formed a collaborative group to undertake a prospectively planned meta-analysis (PPMA) of such studies [44]. Other investigators interested in collaborating in such a PPMA should contact this trial team directly.

### Current trial status

Recruiting.

## Additional files

Additional file 1: Checklist: recommended items to address in a clinical trial protocol and related documents*. (DOC 121 kb)

Additional file 2: Diagnostic criteria (UK Working Party Diagnostic Criteria for Atopic Dermatitis). (DOCX 11 kb)

Additional file 3: The Eczema Area and Severity Index (EASI); clinician-reported signs severity scale. (DOCX 15 kb)

Additional file 4: The Patient Oriented Eczema Measure (POEM); patient-reported symptoms severity scale. (DOCX 1521 kb)

## Abbreviations

AE: Atopic eczema
BEEP: Barrier Enhancement for Eczema Prevention
CHU-9D: Child Health Utility 9D
CRN: Clinical Research Network
EASI: Eczema Area and Severity Index
EQ-5D-5L: EuroQol-5D-5L
*FLG*: Gene encoding filaggrin
GP: General Practitioner
HOME: Harmonising Outcome Measures for Eczema
HTA: Health Technology Assessment
NCTU: Nottingham Clinical Trials Unit
NHS: National Health Service
NICE: National Institute for Health and Care Excellence
NIHR: National Institute for Health Research
NRES: National Research Ethics Service
POEM: Patient-oriented eczema measure
PPMA: Prospectively planned meta-analysis
QALY: Quality-adjusted life year
SWAT: Studies Within A Trial
TSC: Trial Steering Committee
UK DCTN: UK Dermatology Clinical Trials Network
