# Supplementary material for: Effectiveness and cost-effectiveness of daily all-over-body application of emollient during the first year of life for preventing atopic eczema in high-risk children (The BEEP trial): protocol for a randomised controlled trial
Source: Trials. 2017 Jul 21;18:343. doi: 10.1186/s13063-017-2031-3 (PMC5521124; doi:10.1186/s13063-017-2031-3)
Supplement: Supplementary file 2 — Diagnostic criteria (UK Working Party Diagnostic Criteria for Atopic Dermatitis). (DOCX 11 kb) [file 13063_2017_2031_MOESM2_ESM.docx]

**The UK refinement of the Hanifin and Rajka diagnostic criteria for atopic eczema**

Must have had an itchy skin condition in the last 12 months

**Plus three or more of:**

i. Onset below age 2*

ii. History of flexural involvement

iii. History of a generally dry skin

iv. history of atopic disease in a first degree relative

v. visible flexural dermatitis as per photographic protocol

* not included because all children are under 4 years of age

The U.K. Working Party's Diagnostic Criteria for Atopic Dermatitis. III. Independent hospital validation. Br J Dermatol. 1994;131(3):406-16.
