# Supplementary material for: Effectiveness and cost-effectiveness of daily all-over-body application of emollient during the first year of life for preventing atopic eczema in high-risk children (The BEEP trial): protocol for a randomised controlled trial
Source: Trials. 2017 Jul 21;18:343. doi: 10.1186/s13063-017-2031-3 (PMC5521124; doi:10.1186/s13063-017-2031-3)
Supplement: Supplementary file 3 — The Eczema Area and Severity Index (EASI); clinician-reported signs severity scale. (DOCX 15 kb) [file 13063_2017_2031_MOESM3_ESM.docx]

**Eczema Area and Severity Index (EASI)**

| **Body Area** | **% Area Affected by Eczema**  **(Tick one only)** | | | | **Criteria** | **Score**  **(Tick one only)** | | | | | |
| --- | --- | --- | --- | --- | --- | --- | --- | --- | --- | --- | --- |
|  |  |  |  |  |  | **Absent (0)** | **Mild (1)** | **(1.5)** | **Moderate (2)** | **(2.5)** | **Severe (3)** |
| **Head and Neck** | Nil |  | 50-69% |  | Erythema |  |  |  |  |  |  |
|  | 1-9% |  | 70-89% |  | Oedema/Papulation |  |  |  |  |  |  |
|  | 10-29% |  | 90-100% |  | Excoriation |  |  |  |  |  |  |
|  | 30-49% |  |  |  | Lichenification |  |  |  |  |  |  |
|  |  |  |  |  |  |  |  |  |  |  |  |
| **Upper Limbs** | Nil |  | 50-69% |  | Erythema |  |  |  |  |  |  |
|  | 1-9% |  | 70-89% |  | Oedema/Papulation |  |  |  |  |  |  |
|  | 10-29% |  | 90-100% |  | Excoriation |  |  |  |  |  |  |
|  | 30-49% |  |  |  | Lichenification |  |  |  |  |  |  |
|  |  |  |  |  |  |  |  |  |  |  |  |
| **Trunk** | Nil |  | 50-69% |  | Erythema |  |  |  |  |  |  |
|  | 1-9% |  | 70-89% |  | Oedema/Papulation |  |  |  |  |  |  |
|  | 10-29% |  | 90-100% |  | Excoriation |  |  |  |  |  |  |
|  | 30-49% |  |  |  | Lichenification |  |  |  |  |  |  |
|  |  |  |  |  |  |  |  |  |  |  |  |
| **Lower Limbs** | Nil |  | 50-69% |  | Erythema |  |  |  |  |  |  |
|  | 1-9% |  | 70-89% |  | Oedema/Papulation |  |  |  |  |  |  |
|  | 10-29% |  | 90-100% |  | Excoriation |  |  |  |  |  |  |
|  | 30-49% |  |  |  | Lichenification |  |  |  |  |  |  |
