# Supplementary material for: Effectiveness and cost-effectiveness of daily all-over-body application of emollient during the first year of life for preventing atopic eczema in high-risk children (The BEEP trial): protocol for a randomised controlled trial
Source: Trials. 2017 Jul 21;18:343. doi: 10.1186/s13063-017-2031-3 (PMC5521124; doi:10.1186/s13063-017-2031-3)
Supplement: Supplementary file 4 — The Patient Oriented Eczema Measure (POEM); patient-reported symptoms severity scale. (DOCX 1521 kb) [file 13063_2017_2031_MOESM4_ESM.docx]

POEM for proxy completion (e.g by parent)

Patient Details: _________________________

______________________________________

______________________________________

____________________________________ __ Date: _________________________________

**Please circle one response for each of the seven questions below about your child’s eczema. If your child is old enough to understand the questions then please fill in the questionnaire together. Please leave blank any questions you feel unable to answer.**

**1. Over the last week, on how many days has your child’s skin been itchy because of their eczema?**

**No days 1-2 days 3-4 days 5-6 days Every day**

**2. Over the last week, on how many nights has your child’s sleep been disturbed because of their eczema?**

**No days 1-2 days 3-4 days 5-6 days Every day**

**3. Over the last week, on how many days has your child’s skin been bleeding because of their eczema?**

**No days 1-2 days 3-4 days 5-6 days Every day**

**4. Over the last week, on how many days has your child’s skin been weeping or oozing clear fluid because of their eczema?**

**No days 1-2 days 3-4 days 5-6 days Every day**

**5. Over the last week, on how many days has your child’s skin been cracked because of their eczema?**

**No days 1-2 days 3-4 days 5-6 days Every day**

**6. Over the last week, on how many days has your child’s skin been flaking off because of their eczema?**

**No days 1-2 days 3-4 days 5-6 days Every day**

**7. Over the last week, on how many days has your child’s skin felt dry or rough because of their eczema?**

**No days 1-2 days 3-4 days 5-6 days Every day**

**Total POEM Score (Maximum 28):** _________________

POEM for proxy completion (e.g by parent)

**References**

Charman CR, Venn AJ, Williams HC. The Patient-Oriented Eczema Measure: Development and Initial Validation of a New Tool for Measuring Atopic Eczema Severity From the Patients' Perspective.

Arch Dermatol. 2004;140:1513-1519

Charman CR, Venn AJ, Ravenscroft JC, Williams HC. Translating Patient-Oriented Eczema Measure (POEM) scores into clinical practice by suggesting severity strata derived using anchor-based methods.

Br J Dermatol. Dec 2013; 169(6): 1326–1332.

**What does a poem score mean?**

To help patients and clinicians to understand their POEM scores, the following bandings have been established (see references below):

- 0 to 2 = Clear or almost clear
- 3 to 7 = Mild eczema
- 8 to 16 = Moderate eczema
- 17 to 24 = Severe eczema
- 25 to 28 = Very severe eczema

**Do I need permission to use the scale?**

Whilst the POEM scale is protected by copyright, it is freely available for use and can be downloaded from: www.nottingham.ac.uk/dermatology

We do however ask that you register your use of the POEM by e-mailing cebd@nottingham.ac.uk with details of how you would like to use the scale, and which countries the scale will be used in.

**How is the scoring done?**

Each of the seven questions carries equal weight and is scored from 0 to 4 as follows:

No days = 0

1-2 days = 1

3-4 days = 2

5-6 days = 3

Every day = 4

**Note:**

• If one question is left unanswered this is scored 0 and the scores are summed and expressed as usual out of a maximum of 28

• If two or more questions are left unanswered the questionnaire is not scored

• If two or more response options are selected, the response option with the highest score should be recorded
